# Translational metagenomics and the human resistome: confronting the menace of the new millennium

**DOI:** 10.1007/s00109-016-1478-0

**Published:** 2016-10-20

**Authors:** Matthias Willmann, Silke Peter

**Affiliations:** 1 Institute of Medical Microbiology and Hygiene, University of Tübingen, Elfriede-Aulhorn-Str. 6, 72076 Tuebingen, Germany; 2 German Center for Infection Research (DZIF), partner site Tübingen, Tübingen, Germany

**Keywords:** Antimicrobial resistance, Antibiotic selection pressure, Next generation sequencing, Clinical diagnostics, Bacteriophages, Public health

## Abstract

The increasing threat of antimicrobial resistance poses one of the greatest challenges to modern medicine. The collection of all antimicrobial resistance genes carried by various microorganisms in the human body is called the human resistome and represents the source of resistance in pathogens that can eventually cause life-threatening and untreatable infections. A deep understanding of the human resistome and its multilateral interaction with various environments is necessary for developing proper measures that can efficiently reduce the spread of resistance. However, the human resistome and its evolution still remain, for the most part, a mystery to researchers. Metagenomics, particularly in combination with next-generation-sequencing technology, provides a powerful methodological approach for studying the human microbiome as well as the pathogenome, the virolume and especially the resistome. We summarize below current knowledge on how the human resistome is shaped and discuss how metagenomics can be employed to improve our understanding of these complex processes, particularly as regards a rapid translation of new findings into clinical diagnostics, infection control and public health.

## Introduction

**Fig. 1:**
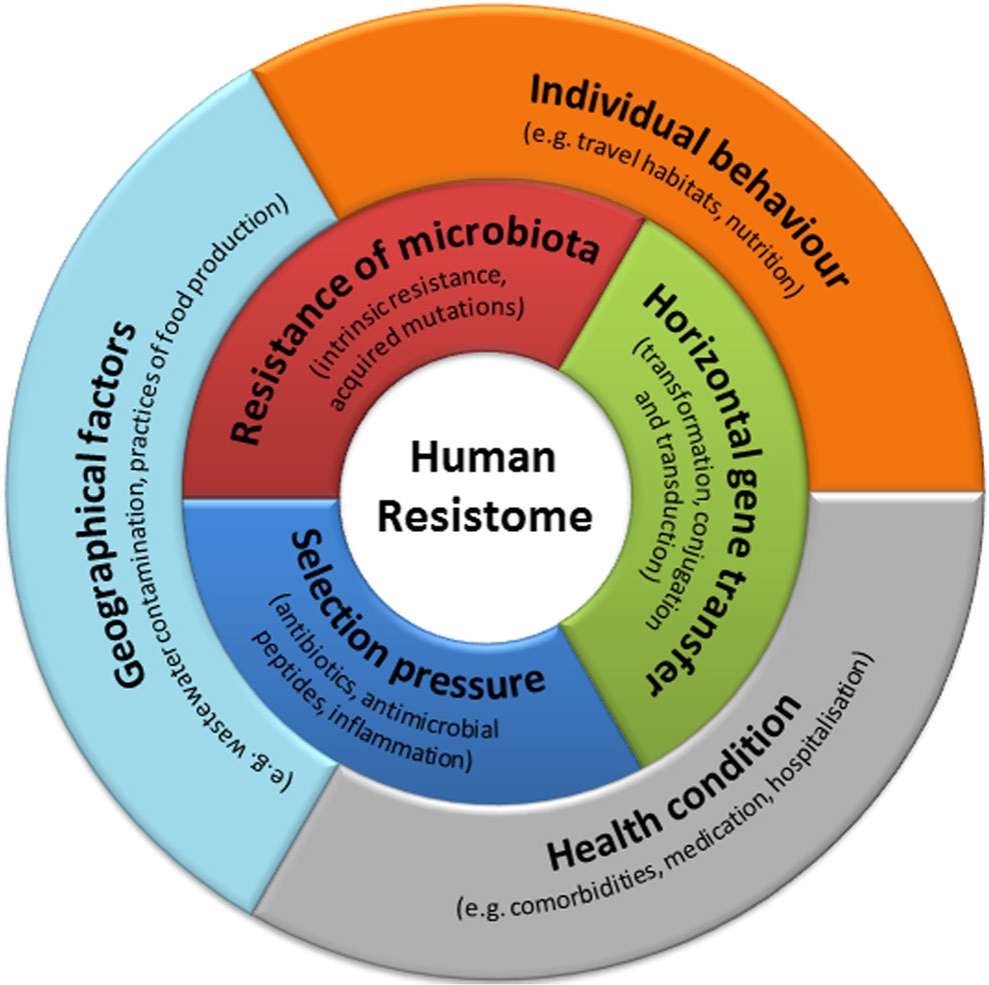
Potential factors shaping the human resistome

The increasing numbers of infections caused by multi-drug resistant (MDR) bacteria have developed into a global and acute public health crisis [1]. The implementation of antimicrobial stewardship programs as well as the development of novel anti-infectives has become a primary focus of public health and research activities in order to reduce the burden of infections caused by MDR pathogens [2]. New mechanisms mediating antimicrobial resistance against antibiotics in the “last line of defence” are steadily reported from all over the world, leaving us only limited therapeutic options for treating serious infections [3]. It is important to understand that human beings can be the vessel in which antimicrobial resistance is born or by which it is spread. The human body harbours at various sites a complex microbial ecosystem and represents a vast reservoir for antimicrobial resistance genes (ARGs), referred to in their totality as the human “resistome” [4]. This human resistome is an open and dynamic entity, shaped by several extrinsic and intrinsic factors that massively interact with each other [5–14] (Fig. 1). To comprehend these interactions, researchers must utilize the full spectrum of modern technology available in our scientific arsenal, and they must start with the study of the bacterial, viral, parasitic and fungal genetic material that is derived from human body sites. Such a methodological approach is termed metagenomics.

In this review, we present an overview of current knowledge on the human resistome. We also outline how metagenomics may be used to characterize the human resistome and how newly acquired insights can be rapidly transferred to clinical microbiology and infection control for practical implementation. We call this approach translational metagenomics.

## Functional metagenomics and the human resistome

Conventional approaches used to determine antimicrobial resistance in bacteria usually rely on a culture-based methodology and focus almost exclusively on human pathogens. Since only a minority of bacterial organisms can be cultured [15–17], and since most of these non-pathogenic commensals are thought to constitute an important resistance gene pool that can be transmitted to human pathogens [18], we need to extend the methodological spectrum for detection of ARGs. Non-culture-based approaches such as metagenomics can overcome these common limitations [19, 20]. Two major techniques used thus far include both functional and sequence-based metagenomics.

The primary strength of functional metagenomics is its ability to detect novel and highly divergent ARGs on a large scale [21]. New ARGs have been discovered every year in the last decade, reflecting a relentless global evolution of a magnitude we have yet to comprehend. Therefore, efforts to detect novel ARGs must be one of the major goals of research if we are to limit the danger of resistance. Our knowledge here can serve as the fundamental basis for developing efficient countermeasures.

In functional metagenomics, DNA is extracted from a material of interest and fragments are cloned into vectors that differ in terms of the feasible insert size of the DNA fragment [22]. Vectors like the pUC family plasmids accept inserts of only small sizes (<10 kb) but do provide a high copy number while using the host’s transcription and translation system. Other vectors like fosmids (25–45 kb inserts), cosmids (15–40 kb inserts) or bacterial artificial chromosomes (100–200 kb inserts) can harbour larger fragments, allowing for a more detailed investigation of the ARG’s genomic environment, e.g. in determining species origin or vicinity to mobile genetic elements. However, such vectors exist in lower copy numbers, and gene recognition by the host organism can be inefficient [23]. The metagenomic library is then transformed into an expression host such as *Escherichia coli*. The host is cultured on selective media and the inserts of antibiotic resistant clones are sequenced either by using the Sanger or next-generation sequencing method. The genes thus identified can subsequently be associated with the observed phenotype [21, 24].

Functional metagenomics has been applied in several informative studies that investigated the composition of the human resistome. Sommers et al. isolated DNA from faeces and saliva of two healthy individuals [25]. Identified ARGs from the mostly anaerobic microflora had only low similarity to known ARGs from pathogenic isolates (54.9 % at amino acid level), while 95 % of ARGs from an aerobic cultured subset were over 90 % identical to known ARGs from pathogens, suggesting a genetic barrier between commensals and pathogens. These results are astonishing since it is thought that the spread of ARGs between bacterial species is mainly driven by a process called horizontal gene transfer (HGT). HGT primarily occurs through the exchange of conjugated plasmids (conjugation) or the exchange of genetic material by bacteriophages (transduction) [26]. The results from Sommers at al. indicate a relatively low HGT frequency. However, both volunteers in the study had not received antibiotics for at least 1 year, a fact that must be taken into consideration when interpreting these results.

Another study offering insights into the development of the gut resistome investigated metagenomic libraries from faecal DNA of healthy infants and children with almost no history of antibiotic exposure [27]. Functional analysis for resistance to 18 antibiotics was performed and revealed a diverse spectrum of novel as well as already documented ARGs in the guts of the infants, even in the absence of selective forces. Of note was the observation of the genes TetX1 and TetX2 that are suspected to confer resistance to tigecycline, which is considered to be a reserve antibiotic agent. The abundance of ARGs in the guts of the infants points to an early establishment of the human gut resistome, possibly supported by a transfer of ARG-harbouring species directly from the mother to the child in combination with early contact to external non-human sources of resistance. A pooled metagenomic library from 22 antibiotic-naïve, 6-month-old infants has confirmed these results by revealing the finding of various aminoglycoside and β-lactam resistance genes with a high homology to commensals’ ARGs [28].

Apart from the extensively studied faecal resistome, the oral microbiota also acts as an important pool of ARGs and is a primary gateway for the acquisition of resistant bacteria [29, 30]. In one study, metagenomes from faeces as well as oral specimens were analysed to determine the frequency distribution of tetracycline and erythromycin resistance genes. The results revealed that *tet(M)* was frequent in the oral microbiota while *erm* genes were present at much lower quantities [31]. Another metagenomic library constructed from human oral cavity samples contained *tet37*, a novel tetracycline resistance gene [32]. The same group of researchers applied functional metagenomics in studying samples of the oral microbiota from 60 adult volunteers [33], detecting amoxicillin and tetracycline resistance elements. The authors though did not provide information about history of antibiotic use. The influence of antibiotics on the oral resistome is still a matter of debate. Clemente et al. found 28 ARGs in the oral microbiome of an antibiotic-naïve Amerindian population, including genes conferring resistance to third-generation cephalosporins [34]. Most of these ARGs exhibited a high similarity (>95 %) to genes in the Human Microbiome Project, which largely contains data from populations that are regularly exposed to commercial antibiotics. Whether the influence of non-anthropogenic antibiotics or other factors could explain this phenomenon remains unclear at this point.

Despite the great methodological advantages of functional metagenomics, some serious limitations exist as well [35, 36]: (i) The bacterial host must fit to the experimental setting. Screening for a certain antibiotic resistance is only possible if the host is not intrinsically resistant to the antibiotic. (ii) Results fundamentally depend on the host’s ability to express the cloned genes. Using only one bacterial host, as found in the majority of studies (mostly *E. coli*), might thus lead to a fairly underrepresented picture of the human resistome. (iii) The use of different media and incubation conditions make a direct comparison between studies difficult. (iv) Accurate quantification of ARGs is not possible. This can hamper the investigation of antibiotic influences, e.g. in determining selection pressure. (v) The choice of the insert size is an important factor. Resistance to antibiotics might be encoded by multiple genes rather than single genes, and short inserts are for the most part not able to provide such a degree of complexity, while larger inserts can have low levels of expression. (iv) Finally, the functional metagenomics approach is labour-intensive and, at present, does not provide the option of high-throughput measurements.

## Sequence-based metagenomics and the human resistome

As with functional metagenomics, the sequence-based approach involves extracting DNA directly from human, animal or environmental samples, including those from unculturable microorganisms. According to their sizes, eukaryotic cells, bacteria and viruses can be separated using filtration or centrifugation before genomic DNA is extracted. Subsequently, the once time-consuming procedure of genomic DNA sequencing is carried out. The development of next-generation sequencing technologies over the past 10 years has dramatically increased the sequencing capabilities and thereby opened a new portal to sequence-based research [37]. The application of shotgun metagenomics allows the generation of millions of DNA or RNA sequences from a sample without the need for prior amplification of a specific target gene [38]. This forms the basis for an unbiased determination of the microbial and functional composition of specimens from any human body site as well as for the characterization and quantification of the respective resistome [38–41]. Sequencing is followed by mapping the metagenomic reads against a catalogue of known sequences usually from national and international databases. The choice of databases depends on whether a functional or taxonomic analysis has priority. Databases that consist of a collection of ARGs include CARD [42], ARG-ANNOT [43], ARDB [44], RED-DB (http://www.fibim.unisi.it/REDDB/), ResFinder [45] or Resfams [46]. Reads that map with a high degree of confidence against specific genes like ARGs can then be counted and thus quantified. Alternatively, reads can be assembled into contigs, and the function of identified genes can be predicted by comparing them with database sequences that have functional annotations. A general comparison between functional and sequence-based metagenomics is presented in Table 1.

**Table 1:** Comparison between functional and sequence-based metagenomics

Functional metagenomics	
Advantages	Disadvantages
• Detection of unknown antimicrobial resistance genes possible • Immediate association between identified gene and resistance phenotype • Detailed investigation of the genomic environment is highly accurate (with large insert fragments)	• Results depend on the host’s ability to express a gene • Intrinsic resistance of the host reduces the spectrum of screening for resistance genes • Study results can be hard to compare due to different media and experimental conditions • Short insert sizes can result in underestimation of a complex mechanism of resistance encoded by multiple genes • Relatively low-throughput
Sequence-based metagenomics	
Advantages	Disadvantages
• Mapping of sequence reads to resistance databases allows to detection of even low abundance ARGs • Several methods for ARG abundance calculation exist as a requirement for determination of selection pressure • Relatively low time-consuming methodology, suitable for high throughput experiments • Raw data can be re-analysed after database updates and allow thus the detection of “novel” ARGs • Beside a resistome analysis, it is possible to analyse the taxonomic and metabolic composition of a sample all at once	• ARGs highly dissimilar to sequences in databases cannot be detected (high database dependency) • No functional confirmation that a gene would actually confer the predicted resistance, only a reflection of its potential • Investigation of the genomic environment of an ARG is less reliable since usually based on a metagenomic assembly that is still not a standardized procedure • Short reads might accidently align to ARGs when using less stringent mapping parameters • No standard criteria for pre-analytics, DNA extraction, sequencing and bioinformatic analysis available

*ARG* antimicrobial resistance gene

Sequence-based metagenomics has been applied in several studies that investigated the structure and development of the human bacterial resistome. An analysis of a human dental plaque revealed that 2.8 % of all predicted genes from the metagenome assembly encoded for resistance to antibiotic or toxic compounds, an astonishingly high number that suggests that the oral cavity is indeed an important reservoir for ARGs [47].

The intestinal resistome, as opposed to the resistomes from other body parts, has been studied extensively. Forslund et al. conducted a comprehensive study by analysing 252 intestinal metagenomes from the USA, Denmark and Spain [48]. ARGs were identified by using the ARDB reference collection [44] after manual augmentation. The authors defined the term “antibiotic resistance potential” as a measure that was based on the abundance of resistance genes relative to the genetic material from species where resistance genes were identified. The antibiotic resistance potential (ARP) revealed geographic differences, with Spain having the highest mean ARP in the majority of antibiotics. However, these data largely indicate a trend, as the Spanish ARP interquartile range overlapped for the most part with the ARPs from the US and Denmark, making it difficult to draw a final conclusion from these results. Interestingly, ARPs for antibiotics that are approved for animal use by the U.S. FDA were significantly higher when compared to antibiotics not approved for animal use, suggesting that the administration of antibiotics in animals promotes the development of resistance in the human intestinal microbiota. Including additional samples from France and Italy revealed that the country-specific ARPs correlated with higher outpatient antibiotic usage in Europe, but the application of different sequencing strategies in the individual country datasets presents too much of a technical challenge for any fair comparison to be made. The same methodology was used in another survey, revealing that ARPs of Chinese samples were even higher for many antibiotics than the Western samples [11]. These results were confirmed by Hu et al., who found 1093 ARGs in the intestinal metagenomic samples of 162 individuals, with the highest relative abundance of ARGs in the Chinese dataset, followed by the Danish and Spanish datasets [40]. A neighbourhood-joining tree based on SNPs in antibiotic resistance genes clustered the Danish and Spanish population but separated the Chinese samples, probably reflecting distinct gene variants attributed to differences in the microbiota and in the antibiotic usage of the different populations.

It is noteworthy that, apart from being localized in the bacterial genome, ARGs might be incorporated in the genome of bacteriophages. Bacteriophages are DNA and RNA viruses that infect bacteria and form a major part of the human gut virome [49]. Bacteriophages act as vehicles for horizontal gene transfer between bacteria via a process referred to as transduction. In comparison to plasmids, bacteriophages can persist in the environment, and the transfer of genetic material does not require the donor bacterium and recipient bacterium to be present in the same environment at the same time. This has led to the hypothesis that bacteriophages might frequently act as vehicles for resistance transfer and are independent reservoirs for antimicrobial resistance [50–54]. Bacteriophages can be encountered everywhere in the environment and are highly abundant in water, coastal sediments and soil samples [55].

In the human gut, virus-like particles (VLPs) including bacteriophages seem to be prevalent in a number comparable to the amount of bacteria at this site. The analysis of VLPs in human faeces from five individuals indicated levels of approximately 10^8^–10^9^ VLPs per gramme faeces [56]. ARGs in bacteriophages can be detected by quantitative real-time PCR ([6, 9, 57]), as demonstrated in a study in which the presence of clinically relevant resistance genes was determined in the bacteriophage fraction of stool samples from 80 healthy individuals. In 77 % of the samples, at least one ARG could be detected. The ß-lactamases TEM and CTX-M-1 genes were the most abundant [58].

Sequence-based metagenomics allows an even broader range of phage-derived ARGs to be detected, given we have a successful and specific extraction of viral DNA prior to sequencing. A broad range of ARGs was detected at different time points in human intestinal viral metagenome sequences from six and five individuals, respectively. ARGs included multidrug efflux transporters, tetracycline and vancomycin resistance genes, and ß-lactamases [59, 60].

Viruses from other body sites also seem to contribute significantly to the human resistome. ARGs mediating resistance to ß-lactams, vancomycin, aminoglycosides, macrolides, tetracyclines and chloramphenicol were detected in viral metagenome sequences from the saliva of five individuals sampled at different time points [60]. Another analysis of five metagenomic virome datasets of sputum samples from cystic fibrosis patients found evidence for an increased abundance of various ARGs when compared to datasets of individuals without disease [61]. In a recent study, the human skin virome of 16 healthy volunteers was analysed at two time points. The isolated VLPs contained 29 unique ARG groups relating to ß-lactamases, efflux pumps, rifampicin and tetracyclines resistance. Approximately half of the identified ARGs were located on contigs that additionally contained phage-associated genes [62].

The data available on the human virome and resistance genes in VLPs are based on only a few studies with small sample sizes. We need more information on phage-host interaction in order for us to understand the mechanisms and dynamics of ARG transfer between commensals and pathogens and how much phage activity does contribute to the shaping of the human resistome, particularly under antibiotic treatment.

While sequence-based metagenomics is a promising approach, particularly in determining the abundance of ARGs, it also has the following limitations: (i) Functional metagenomic studies showed a high number of ARGs with no significant similarity to sequenced genes deposed in databases. Such novel sequences cannot easily be identified using sequence-based metagenomics, and the resistance potential of a sample might be severely underestimated. (ii) Functional confirmation in terms of whether a gene is expressed and can actually confer resistance or whether it simply encodes an alternative function in the given host is not provided. Metatranscriptomics or proteomics might provide additional information [22, 63, 64], but these should be combined with DNA sequencing, as the actual protein expression could simply indicate a momentary appearance. Of note, some degree of variation can also be expected in DNA sequencing [65]. (iii) Mapping of reads to reference sequences usually provides only limited information on the genetic environment of the ARG. However, information about the host species, or whether a gene is located on a mobile genetic element, can offer important insights regarding the clinical relevance of an ARG. A solution could be a metagenomic assembly of the sample, but there are no standard criteria to assess the success of such a complex analytical process [66–69].

Despite these limitations, the application of sequence-based metagenomics offers a wide range of possibilities in diagnostics, infection control and research since the technology is capable of exploring the taxonomic composition, ARG abundance and the functional profile of a sample all at once.

## Translational metagenomics in clinical diagnostics

Sequence-based metagenomics in clinical microbiology diagnostics is seen as a method offering great potential for the future, not only for the examination of the human resistome but also for the detection of human pathogens, among others. The approach does not require a priori knowledge of the expected pathogen. It enables the detection of co-infections [70] and can provide information on the host response in the case of RNA sequencing [71]. Several reports describing the use of sequence-based metagenomics to resolve cases involving unclear infectious diseases have demonstrated the advantages of the method for clinical diagnostics [72–76]. In addition, the technique can be applied in outbreak situations to identify the causative agent [77, 78], including novel infectious agents [79], while also providing phylogenetic information on the organisms involved in infections [72, 80].

Besides identifying pathogens, appropriate patient management depends on correct and fast antimicrobial susceptibility testing results. Despite being promising, only a few studies have used sequence-based metagenomics to identify and characterize the resistance pattern of a pathogen. One study compared RNA metagenomic sequencing with conventional diagnostics in influenza-positive respiratory specimens from 24 patients. The presence of resistance markers in the hemagglutinin (HA) and neuraminidase gene was evaluated in a subset of 6 samples. Mutations that are associated with medium resistance to zanamivir and with resistance to adamantane were detected in the neuraminidase gene. All strains were predicted to be susceptible to oseltamivir and peramivir due to the absence of known resistance markers [70]. The utility of sequence-based metagenomics in this context is supported by Graf et al., who compared metagenomics RNA sequences obtained from paediatric nasopharyngeal swabs with a commercial respiratory PCR panel. Sufficient sequencing coverage of the neuraminidase gene was obtained in 6 out of 8 H1N1 influenza-positive samples. The oseltamivir resistance marker H275Y was not detected in any of the six samples [81]. In a study conducted in South Africa, the authors were able to determine the frequency of minor variant HIV drug resistance mutations in infected newborns [82].

Sequence-based metagenomics has also been applied to detect bacterial ARGs in clinical settings. Conventional microbiological culture was compared to whole-genome sequencing (WGS) of bacterial isolates and sequence-based metagenomics from 35 urine samples [83]. The predicted resistance phenotype obtained from WGS data and metagenomics data correlated surprisingly well with the conventional antimicrobial resistance pattern of the isolates. However, in two samples, more ARGs were obtained by metagenomics compared to the WGS dataset. In addition, ARGs were also present in two of four culture-negative samples, most likely originating from urinary tract flora or unculturable organisms.

The investigation of *Clostridium difficile* positive stool samples and five stool samples from healthy controls revealed that class A Beta-lactamase genes and tetracycline ARGs were most abundant, followed by macrolide ARGs [84]. However, the authors could not determine the bacterial species from which the ARGs originated, which limits the value of these results. In another study, investigating the utility of sequence-based metagenomics in patients suffering from *Mycobacterium tuberculosis* infections, eight smear-positive sputum samples were analysed. In seven samples, metagenomic sequencing data allowed the identification of the species and linage within the *M. tuberculosis* complex. However, no prediction of the susceptibility patterns of the Mycobacteria could be drawn from the data, most likely due to the insufficient coverage depth of *M. tuberculosis* in the sputum sample [85].

A recent study used sequence-based metagenomics to detect free-circulating DNA from plasma samples of septic patients [86]. The authors analysed 62 samples from seven sepsis patients, six patients who underwent abdominal surgery and 12 healthy volunteers at different time points. Quantitative evaluation of non-human reads and the introduction of a sepsis indicating quantifier score enabled the identification of the sepsis-causing agents. The results matched perfectly with the organisms recovered from the blood cultures in these patients. In one patient who suffered from sepsis after liver transplantation, *Enterococcus faecium* reads and hits for the resistance genes *vanB*, *vanS*_*B*_, *tetl* and *sta4* were identified. A vancomycin-resistant *E. faecium* could be recovered from the patient’s blood cultures, demonstrating the usefulness of the methodology, particularly in sepsis, which is often caused by a single bacterium, a situation that increases the likelihood that a detected ARG originates from the respective pathogen.

All reports of clinical application of sequence-based metagenomics are individual cases or small-scale investigations, limited through their size in providing answers to some essential questions. Before implementing sequence-based metagenomics for identifying pathogens and predicting their antimicrobial susceptibility patterns, we need to establish standardized operation procedures for different clinical specimens. Standardization needs to take the following factors into account: (i) the resident flora, (ii) the sequencing depth required to achieve a defined coverage as well as (iii) the length of a sequence read that is needed to determine the taxonomic origin of ARGs. Lastly, the lack of applicable regulatory guidelines for the implementation of sequence-based metagenomics in routine diagnostics hampers its integration in clinical microbiology diagnostics [19], a legal issue that should be resolved in the near future.

## Translational metagenomics in determining antibiotic treatment impact

Some research groups have recently addressed the question of how metagenomics can be used to determine the impact of administering antibiotics on the intestinal resistome in patients or volunteers [39, 41]. Information about the selective force of antibiotics is crucial as it enables clinicians directly to compare different drug regimens and to decide on which drug or drug combination should be preferred over others due to a lower influence on the resistome of their patients. Such strategies applied in antibiotic stewardship programs could offer efficient ways in reducing the spread of multidrug resistance.

Determining selection pressure is fundamentally based on a genuine quantification of ARGs. Several quantification efforts have been applied and published. Buelow et al. investigated the impact of selective digestive decontamination (SDD) on the gut’s resistome in critically ill patients [41]. SDD involved a regime that consisted of colistin, tobramycin, a third-generation cephalosporin and amphotericin B. Under treatment, the authors observed an 6.7-fold increase in the abundance of aminoglycoside resistance genes, particularly for *aph(2″)-Ib* and an *aadE*-like gene. They used a metagenomic assembly strategy for ARG quantification. In a local Blast search, a clustered version of the Resistance Determinants Database (RED-DB; http://www.fibim.unisi.it/REDDB/) was used as a query to find ARG on assembled contigs. A log-transformed quotient of the coverage of a contig encoding an ARG over the average sequencing depth of the total assembly was then determined as a value reflecting the relative abundance. While this is a valid estimation of the ARG content, it is primarily based on the quality of the metagenomic assembly and is likely to overlook ARGs in low quantities, since they usually fail to be assembled. Moreover, the authors of the study have reported issues with small contig size preventing a detailed investigation of the genomic environment of ARGs. Therefore, they have constructed fosmid libraries with inserts of about 40 kb and found ARG sequences associated with phage-, plasmid or IS element structures and with anaerobes from the phyla Firmicutes (*Subdoligranulum, Clostridia*), Bacteroidetes (*Bacteroides uniformis*) and Actinobacteria as species origin. An estimate of the selective force caused by the SDD regime was not calculated.

Recently, our group developed an analysis pipeline to determine selective pressure by using sequence-based metagenomics in two volunteers receiving ciprofloxacin [39]. We compared different ARG abundance calculations, all based on the mapping of 100-bp-long sequence reads to either the Comprehensive Antibiotic Resistance Database (CARD) [42] or a non-redundant protein catalogue consisting of sample specific proteins, RefSeq proteins and CARD. We observed a generally high correlation between the methods. Differences were mostly due to the database used and were less dependent on the calculation and normalization method. Quantification based on sequence reads correlated highly with a targeted quantitative PCR. Both fixed and random effects models were subsequently used specifically to calculate the selection pressure caused by ciprofloxacin on different groups of ARGs. Interestingly, while positive selection was observed for class D beta-lactamases, tetracyclines and macrolides ARGs, there was also a substantial fraction of ARGs that was negatively selected, most likely due to an eradication of species harbouring these genes (e.g. class A beta-lactamases and glycopeptides). Such a mechanism has been demonstrated in an infant cohort that was exposed to antibiotics and whose stool samples were analysed through sequence-based metagenomics. Here, the progression of the abundance of a beta-lactamase gene correlated highly with the relative abundance of *Klebsiella pneumoniae* under treatment. Thus, this species was likely to carry this resistance gene [87]. Quantification based on sequence reads has the advantage that it does not rely on a metagenomic assembly and would thus also count low abundance genes. However, ARG specificity depends on the mapping parameters, and stringent criteria might still result in an underestimation of ARGs.

None of the ARG quantification approaches described above has targeted resistance-conferring mutations within an antibiotic target gene, also called target resistance alleles (TRAs). Resistance to important antibiotics such as quinolones and rifampicin is partly or fully based on TRAs. However, quantification of TRAs is an issue since sequence reads that match to an antibiotic target gene but do not span a TRA region are difficult to assign, as it remains unclear whether they originate from a wild-type or mutated version of the antibiotic target gene. Simply ignoring such sequence reads might result in a severe bias. Field and Hershberg have analysed metagenomic datasets from the Integrated Microbial Genomes with Microbiom Samples database and determined TRA frequency for quinolones (*gyrA*/*parC*), rifamycins (*rpoB*) and streptomycin (*rpsL*) [88]. TRA frequencies for quinolones were found to be high among all human datasets (~40 %), though how much the administration of antibiotics has impacted the results is unclear.

While it is assumed that a positive selection is mostly due to enrichment of resistant species, administering antibiotics might also directly impact the dissemination of ARGs by horizontal gene transfer (HGT), which is the most common mechanism for pathogens to acquire new resistance genes (reviewed in [10]). HGT appears to occur 25-fold more frequently between human-associated bacteria than among ecologically diverse non-human isolates [86]. Whereas conjugation has long been considered of crucial importance for the dissemination of ARGs, recent studies suggest that transduction by bacteriophages might play a more important role than previously recognized [10]; however, data from clinical studies remain limited. Abeles et al. compared the oral and faecal viromes of four untreated healthy individuals and five individuals who received intravenous antibiotics at two time points. Although not significant, there was a trend toward a treatment-induced expansion of ARGs in the faecal viromes, which was not detected in the oral viromes. However, the five patients in the treatment group received different combination therapies composed of cefazolin, ceftazidime, trimethoprim/sulfamethaxole, vancomycin, daptomycin and rifampicin, which made the group very inhomogeneous. Unfortunately, no baseline sample of the patients was obtained before the start of the treatment, limiting the conclusions to be drawn from this study [60].

**Fig. 2:**
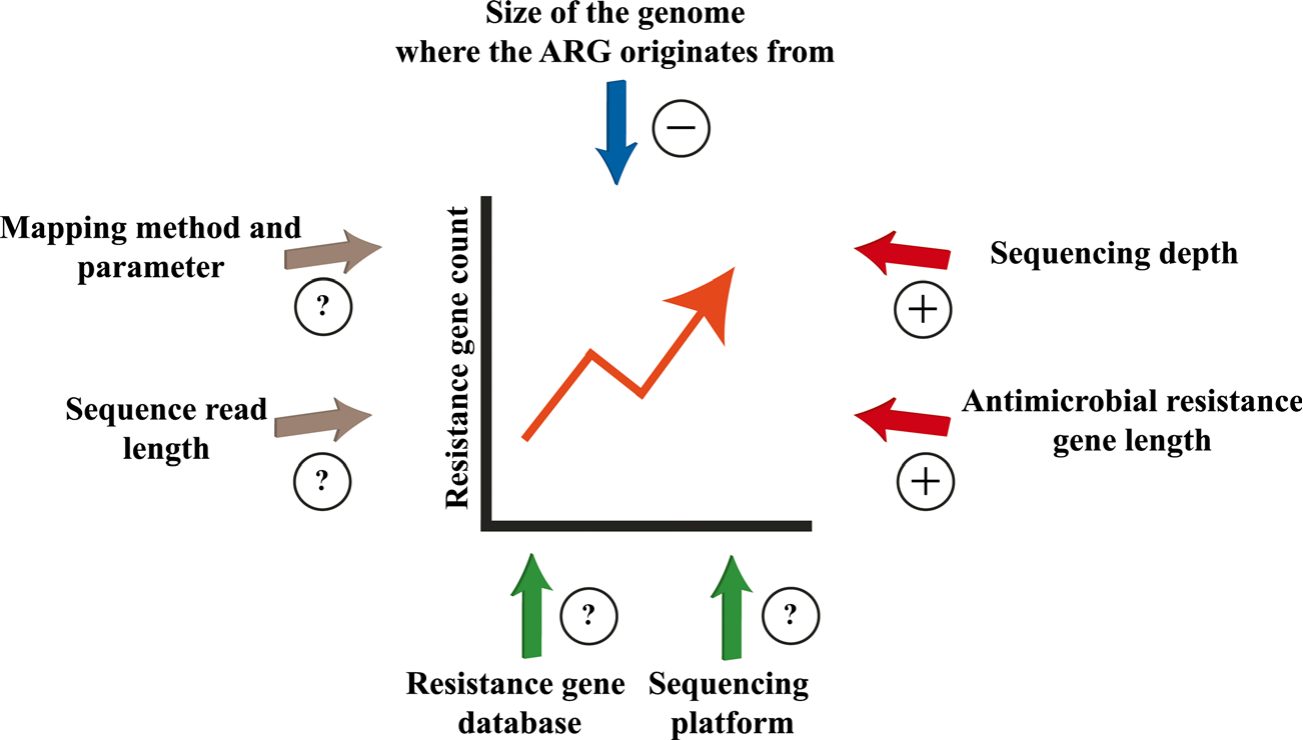
Factors influencing the antimicrobial resistance gene count. Several factors have an impact on the resistance gene count [39, 91, 92]. *Red arrows* indicate a positive correlation, for instance an increase in the antimicrobial resistance gene (ARG) length increases also the ARG count. The *blue arrows* indicates a negative correlation, like the increase in the size of the genome where the ARG is derived from lowers the chance of the ARG to be found in the metagenome. *Green arrows* indicate factors with a proven influence on the resistance gene count; however, the relationship cannot be easily quantified in terms of direction. *Grey arrows* indicate factors which influence on the resistance gene count have not been systematically investigated but are likely to have an impact

These exemplary studies have revealed that important attempts have been made in estimating the impact of antibiotics or in calculating selection pressure. Our goal must be in producing more comparable studies with set standards for determining ARG abundance and selection pressure. This would involve the following aspects: (i) Results can only be compared when similar sequencing strategies and pre-analytical procedures are used. (ii) A critical sequencing depth must be specified to avoid underestimation of low abundant but clinically relevant ARGs. (iii) A consensus must be found regarding a definition of clinically relevant antimicrobial resistant genes. A classification of ARGs according to their attributed risk, which is dependent on their potential to be spread, or to their origin in human pathogens, has already been suggested [89], but needs to be extended for these types of studies. The determination of the human resistome without the availability of valid information on the origin of ARGs or their environment might induce an over-usage of antimicrobials. This problem will be overcome at least in part with the increasing availability of long-range read technologies. For example, a long-read technology has been successfully applied in identifying the chikungunya virus [90]. (iv) Factors that influence ARG counts in metagenomic datasets have been identified (Fig. 2), but no standards exist for the normalization of such counts. While efforts have been made to develop adequate methods [91], data normalization must take into account the impact size of each influencing factor in order to produce an appropriate weighting.

Additionally, since it is known that the composition of the human resistome varies between different countries [11, 40, 48], it can also be assumed that there are considerable variations between different regions or hospitals. It is thus not unlikely that the impact of antibiotics can vary as well and can even be highly dependent on the baseline resistome of an individual or group of individuals in a certain environmental setting. Therefore, therapeutic strategies that would consider antibiotic selection pressure might need to be adapted for the regional resistance epidemiology in a way of a “personalized public health” concept to unleash fully the preventive potential in this fight against resistance.

## A practical and prospective workflow

**Fig. 3:**
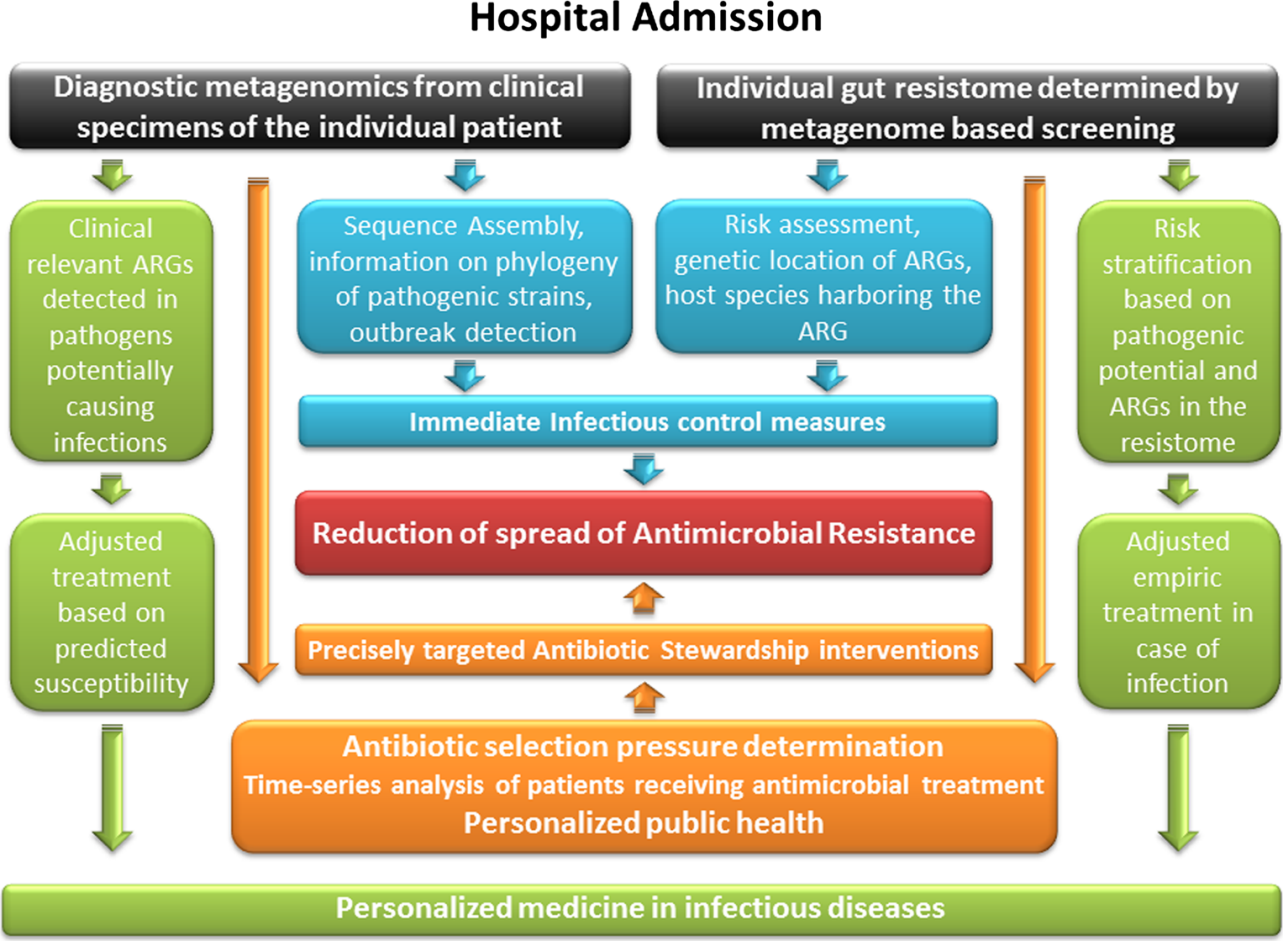
Possibilities for future implementation of metagenomics in infection control and clinical microbiology

The current limitations of translational metagenomic approaches are likely to be overcome in the future. Our contribution to that future is to draft a practical workflow of how metagenomics could be applied in hospitals and how the individual patient, the hospital and the public health system could benefit from it (Fig. 3). At and after admission, sequence-based metagenomics could be used for a routine screening of clinically relevant ARGs in patient stool samples. Relevant resistance in pathogens would lead to appropriate infection control measures. Phylogenetic analyses of pathogen genomes could contribute, when necessary, to outbreak management. Furthermore, in case of a subsequent nosocomial infection, appropriate empirical treatment could be immediately administered.

The initial sample taken at admission can serve as an analytical baseline. In the event that patients receive antibiotics during their hospital stay, the impact of the treatment could be investigated by a time series analysis of further samples. The results would reflect the local situation and could be used to design efficient antibiotic stewardship programs.

Apart from a routine screening process, metagenomics could be applied in patients suspected of having an infection. Pathogens as well as virulence factors, whose presence could indicate the causality between an identified pathogen and the clinical case, could be detected. ARGs on relevant pathogens could be revealed and appropriate treatment promptly delivered.

Such a workflow is likely to result in improved patient treatment, in a more detailed epidemiological analysis of nosocomial pathogens, in faster and potentially more reliable diagnostics, as well as in advances in hospital infection control and thus potentially in a reduction in the spread of resistance.

## Final remarks

Despite the enormous efforts already being made to extend our understanding of the human resistome and its various interactions with the environment, we only have preliminary knowledge available to us for implementing strategies that would efficiently counter the actual threat of resistance in our hospitals. Metagenomic methods offer great potential here, especially if we focus on their clinical applicability, which is the major characteristic of what we call translational metagenomics.
